# Supplementary material for: TopDIA: A Software Tool for Top-Down Data-Independent Acquisition Proteomics
Source: bioRxiv. 2024 Apr 9:2024.04.05.588302. Preprint. [Version 1] doi: 10.1101/2024.04.05.588302 (PMC11030422; doi:10.1101/2024.04.05.588302)
Supplement: Supplement 1 [file media-1.pdf]

Supporting Information

TopDIA: A Software Tool for Top-Down Data-Independent Acquisition Proteomics

Abdul Rehman Basharat[1+], Xingzhao Xiong[2+], Tian Xu[3], Yong Zang[4], Liangliang Sun[3], and Xiaowen Liu[2,*]

[1]Department of BioHealth Informatics, School of Informatics and Computing, Indiana University-Purdue University Indianapolis, Indianapolis, IN, 46202, USA
[2]Deming Department of Medicine, Tulane University School of Medicine, New Orleans, LA, 70112, USA
[3]Department of Chemistry, Michigan State University, East Lansing, MI, 48824, USA
[4]Department of Biostatistics and Health Data Sciences, Indiana University School of Medicine, Indianapolis, IN, 46202, USA

[+]Co-first authors
*Correspondence: Xiaowen Liu (xwliu@tulane.edu); Liangliang Sun (lsun@chemistry.msu.edu)

Table of Content

Supplementary Figures **3**

Supplementary Figure S1. A TD-DIA-MS experiment is divided into cycles, each of which contains an MS1 scan and 20 MS/MS scans. All the cycles in a run are sorted in the increasing order of the retention time and the index of a cycle is its position in the sorted list. 3

Supplementary Figure S2. Extracted ion chromatograms (XICs) of an SCPF and a fragment feature are linear interpolated. Subsequently, the interpolated intensities within the XICs are normalized so that the area under the XIC equals 1. The shared area under the normalized interpolated XICs is reported as the shared XIC of the SCPF and the fragment feature. 3

Supplementary Figure S3. Receiver operating characteristic (ROC) curve of the logistic regression model on the validation SCPF and fragment feature pairs generated from the DIA-TRAIN data.......... 4

Supplementary Tables **5**

Supplementary Table S1. Parameter settings for the modified version of TopFD for extracting proteoform features from TD-DIA-MS data 5

Supplementary Table S2. Parameter settings for the modified version of TopFD for extracting fragment features from TD-DIA-MS/MS data 5

Supplementary Table S3. Parameter settings for TopFD (version 1.7.2) 5

Supplementary Table S4. Variable PTMs used in TopPIC for protein identification 6

Supplementary Table S5. Parameter settings of TopPIC for analyzing TD-DDA-MS and TD-DIA-MS data sets 6

Supplementary Table S6. Comparison of proteoform and protein identifications between DIA-TEST-1 and DDA-TEST-1 6

Supplementary Table S7. Comparison of proteoform and protein identifications between DIA-TEST-2 and DDA-TEST-2 7

Supplementary Table S8. Comparison of proteoforms and proteins identified from DIA-TEST-1 by the pseudo spectra and single spectra approaches 7

Supplementary Table S9. Comparison of proteoform and protein identifications between DIA-TEST-1 and DIA-TEST-2.. 7

Supplementary Table S10. Comparison of proteoform and protein identifications between DDA-TEST-1 and DDA-TEST-2.. 7

Supplementary Figures

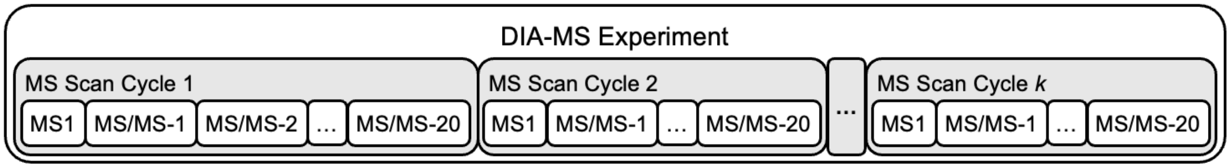

Supplementary Figure S1. A TD-DIA-MS experiment is divided into cycles, each of which contains an MS1 scan and 20 MS/MS scans. All the cycles in a run are sorted in the increasing order of the retention time and the index of a cycle is its position in the sorted list.

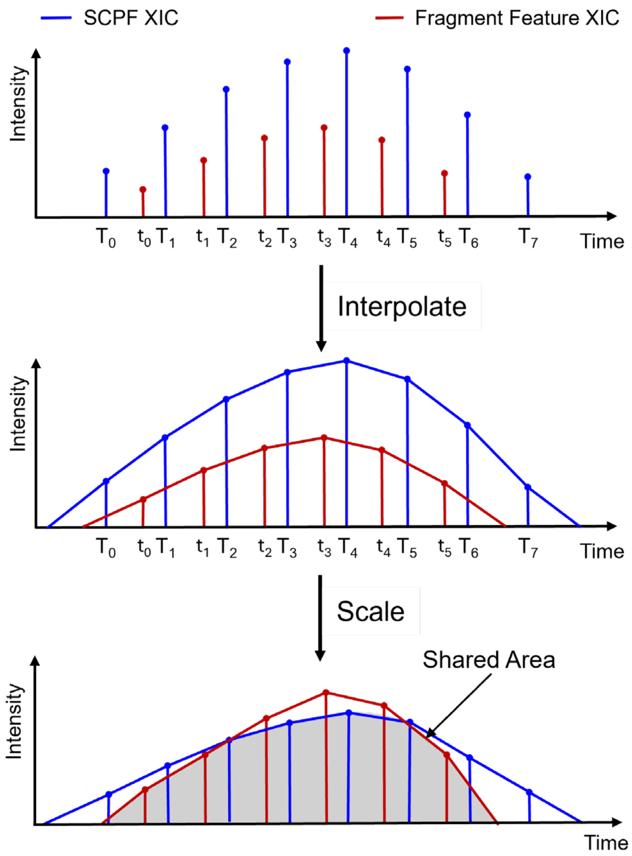

Supplementary Figure S2. Extracted ion chromatograms (XICs) of an SCPF and a fragment feature are linear interpolated. Subsequently, the interpolated intensities within the XICs are normalized so that the area under the XIC equals 1. The shared area under the normalized interpolated XICs is reported as the shared XIC of the SCPF and the fragment feature.

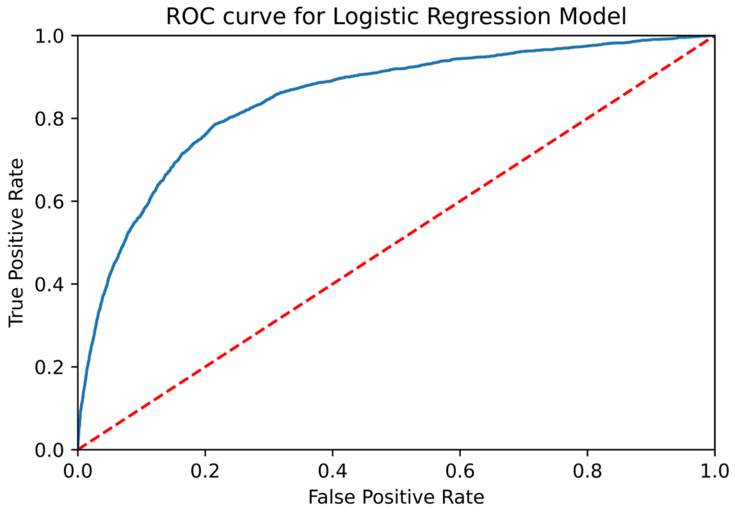

Supplementary Figure S3. The receiver operating characteristic (ROC) curve of the logistic regression model on the validation SCPF and fragment feature pairs generated from the DIA-TRAIN data.

Supplementary Tables

Supplementary Table S1. Parameter settings for the modified version of TopFD for extracting proteoform features from TD-DIA-MS data

Parameter	Value
Maximum charge	60
Maximum mass	70,000 Da
MS1 signal noise ratio in MS-Deconv	3.0
M/z error tolerance in MS-Deconv	0.02
Do final filtering in MS-Deconv	True
Use EnvCNN score in MS-Deconv	True
Use single scan noise level during feature extraction	True
Minimum scan number in features	2
Seed envelope intensity correlation tolerance	0.5
ECScore cutoff	0

Supplementary Table S2. Parameter settings for the modified version of TopFD for extracting fragment features from TD-DIA-MS/MS data

Parameter	Value
Maximum charge	60
Maximum mass	70,000 Da
MS/MS signal noise ratio in MS-Deconv	1.0
Isolation window	4.0 *m/z*
M/z error tolerance in MS-Deconv	0.02
Do final filtering in MS-Deconv	True
Use single scan noise level during feature extraction	True
Minimum scan number in features	1
Seed envelope intensity correlation tolerance	0
ECScore cutoff	0

Supplementary Table S3. Parameter settings for TopFD (version 1.7.2)

Parameter	Value
Maximum charge	60
Maximum mass	70,000 Da
MS1 signal noise ratio in MS-Deconv	3.0
MS/MS signal noise ratio in MS-Deconv	1.0
M/z error tolerance in MS-Deconv	0.02
Do final filtering in MS-Deconv	True
Use EnvCNN score in MS-Deconv	True
Use single scan noise level during feature extraction	True
Isolation window	DDA: 3.0 *m/z*<br>DIA: 4.0 *m/z*
Minimum scan number in features	DDA: 3<br>DIA: 2
Seed envelope intensity correlation tolerance	0.5
ECScore cutoff	0.5
Additional feature search	False

Supplementary Table S4. Variable PTMs used in TopPIC for protein identification

PTM	Mass (Da)	Possible amino acid sites
Methylation	14.015650	CKRHDENQ
Oxidation	15.994915	CPKDNRY
Acetylation	42.010565	Any
Phosphorylation	79.966331	Any

Supplementary Table S5. Parameter settings of TopPIC for analyzing TD-DDA-MS and TD-DIA-MS data sets

Parameter	Value
Fragmentation method	File
Search type	Target+Decoy
N-terminal forms of proteins	NONE, M_ACETYLATION, NME, NME_ACETYLATION
Fixed modifications	No
Maximum number of variable modifications	3
Variable modifications	Methylation, Oxidation, Acetylation, and Phosphorylation
Spectrum level cutoff type for filtering PrSMs	FDR
The cutoff value for filtering PrSMs	0.01
Spectrum level cutoff type for filtering proteoforms	FDR
The cutoff value for filtering proteoforms	0.01
Error tolerance for precursor and fragment masses	10 ppm
Error tolerance for identifying PrSM clusters	1.2 Da
Maximum number of unexpected mass shifts	1
Minimum value of the mass shift	-500 Da
Maximum value of the mass shift	500 Da
E-values computation	Generating function
Use TopFD feature	DDA: True<br>DIA: False

Supplementary Table S6. Comparison of proteoform and protein identifications between DIA-TEST-1 and DDA-TEST-1

m/z range	DIA		DDA		Shared	
	Proteoforms	Proteins	Proteoforms	Proteins	Proteoforms	Proteins
720-800	176	84	173	82	122	72
800-880	199	93	209	91	134	78
880-960	205	98	263	109	164	88
960-1040	202	95	233	101	151	84
1040-1120	192	92	190	77	136	68
1120-1200	166	78	157	73	113	60

Supplementary Table S7. Comparison of proteoform and protein identifications between DIA-TEST-2 and DDA-TEST-2

m/z range	DIA		DDA		Shared	
	Proteoforms	Proteins	Proteoforms	Proteins	Proteoforms	Proteins
720-800	151	72	171	74	108	62
800-880	210	94	175	75	126	67
880-960	213	103	227	94	152	81
960-1040	217	105	225	99	145	80
1040-1120	201	97	225	96	148	79
1120-1200	134	70	178	79	106	59

Supplementary Table S8. Comparison of proteoforms and proteins identified from DIA-TEST-1 by the pseudo spectra and single spectra approaches

m/z range	Pseudo spectra		Single spectra		Shared	
	Proteoforms	Proteins	Proteoforms	Proteins	Proteoforms	Proteins
720-800	176	84	167	76	130	72
800-880	199	93	182	82	144	78
880-960	205	98	211	90	154	83
960-1040	202	95	187	91	153	85
1040-1120	192	92	159	73	134	71
1120-1200	166	78	155	68	126	63

Supplementary Table S9. Comparison of proteoform and protein identifications between DIA-TEST-1 and DIA-TEST-2

m/z range	DIA-TEST-1		DIA-TEST-2		Shared	
	Proteoforms	Proteins	Proteoforms	Proteins	Proteoforms	Proteins
720-800	176	84	151	72	109 (72.2%)	64 (88.9%)
800-880	199	93	210	94	126 (72.0%)	75 (89.3%)
880-960	205	98	213	103	145 (70.7%)	84 (85.7%)
960-1040	202	95	217	105	143 (70.8%)	83 (87.4%)
1040-1120	192	92	201	97	141 (74.2%)	82 (89.1%)
1120-1200	166	78	134	70	104 (77.6%)	63 (90.0%)

Supplementary Table S10. Comparison of proteoform and protein identifications between DDA-TEST-1 and DDA-TEST-2

m/z range	DDA-TEST-1		DDA-TEST-2		Shared	
	Proteoforms	Proteins	Proteoforms	Proteins	Proteoforms	Proteins
720-800	173	82	171	74	115 (76.1%)	66 (94.3%)
800-880	209	91	175	75	145 (82.8%)	73 (97.3%)
880-960	263	109	227	94	161 (78.5%)	80 (86.9%)
960-1040	233	101	225	99	160 (79.2%)	84 (82.3%)
1040-1120	190	77	225	96	158 (83.1%)	74 (96.1%)
1120-1200	157	73	178	79	113 (84.3%)	62 (93.9%)
